# Overcoming Dormancy of Black Locust (*Robinia pseudoacacia* L.) Seeds Using Various Non-Thermal Plasma Sources

**DOI:** 10.3390/plants14050728

**Published:** 2025-02-27

**Authors:** Vladimír Scholtz, Jana Jirešová, Josef Khun, Tomasz Czapka, Jaroslav Julák, Myron Klenivskyi

**Affiliations:** 1 Department of Physics and Measurements, University of Chemistry and Technology Prague, Technická 5, 166 28 Prague, Czech Republic; jiresovj@vscht.cz (J.J.); khunj@vscht.cz (J.K.);; 2 Department of Electrical Engineering Fundamentals, Faculty of Electrical Engineering, Wroclaw University of Science and Technology, Wybrzeże Wyspiańskiego 27, 50-370 Wroclaw, Poland; tomasz.czapka@pwr.edu.pl

**Keywords:** non-thermal plasma, black locust, *Robinia pseudoacacia*, seed dormancy, seed treatment, seed germination

## Abstract

Black locust (Fabaceae family) seeds are known for their strong dormant state and are an excellent candidate for studying and developing methods to break dormancy. We investigated overcoming the dormancy using several different sources of non-thermal plasma, which, by modifying, etching, or disrupting the waxy seed coat, allowed water to penetrate the seeds and initiate germination. All plasma sources tested enhanced seed germination to varying degrees, with over 80% germination observed when using a dielectric barrier discharge, while control seeds showed no germination. Non-thermal plasma treatment significantly decreased the water contact angle of the seed surface from an initial 120° (for untreated seeds) to complete wetting when using a dielectric barrier discharge or atmospheric-pressure plasma jet. The experiments indicate two mechanisms for the modification of the waxy seed coat by a non-thermal plasma: hydrophilization of the wax surface through the binding of oxygen particles and etching of narrow channels in the wax layer, allowing water to penetrate the seed.

## 1. Introduction

### 1.1. Robinia pseudoacacia

*Robinia pseudoacacia*, also known as black locust or false acacia, is a deciduous tree of the legume family *Fabaceae*, tribe *Robinieae*. Although this species is native to certain regions of the United States [1], it has successfully naturalized in temperate regions worldwide, including North America, Europe, Southern Africa, and Asia [2,3]. *Robinia* has been extensively planted for soil erosion control and as a source of hard, durable timber [4,5,6]. Although its bark, leaves, and wood are toxic to humans and animals, its young pods are edible. Black locust seeds have a rich, nutritional profile and are particularly valued for their high protein, fiber, and essential fatty acid content [7,8,9]. Furthermore, black locust seeds and flowers are recognized for their numerous health benefits, including antioxidant properties, anti-inflammatory effects, immune system enhancement, and promotion of the digestive process [10,11,12]. In addition, black locust flowers are also a source of valuable bee honey.

A notable feature of acacia seeds is their hard testa, which provides protection from adverse environmental conditions. On the other hand, due to its hydrophobic properties, the seed coat repels water, preventing it from penetrating the seeds [13], which prevents the seeds from germinating and leaves them dormant for a long period of time [14,15,16]. To break seed dormancy, one needs to disrupt the seed coat using hot water, acid, or mechanical scarification [13,16,17]. An alternative and promising technique for breaking seed dormancy and stimulating their germination is the treatment of seeds with a non-thermal plasma (NTP) [18,19,20]. The use of NTP is a relatively new direction that is rapidly developing and finding its place in various spheres of life.

### 1.2. Non-Thermal Plasma

Plasma, often referred to as the fourth state of matter, is a partially or fully ionized gas. It is typically classified into thermal and non-thermal plasma. A thermal (equilibrium) plasma has electron, ion, and gas temperatures of tens of thousands of kelvins and occurs in the sun, lightning, electric sparks, tokamaks, etc. Due to high ion and gas temperatures, a thermal plasma is not applicable in biology or plant cultivation. In contrast, a non-thermal (non-equilibrium) plasma can have ion and gas temperatures as low as the ambient temperature, allowing it to be applied to living tissues without causing thermal damage, although the electron temperature can be high. Many physical and chemical processes occur in plasma, leading to the formation of various plasma species that give the plasma unique properties. The chemical kinetics occurring in NTP and the mechanisms of NTP impact on various objects are very complex (see, e.g., [21,22,23]).

### 1.3. NTP Generation

Plasma can be generated using any kind of energy that can ionize gases, including electrical, thermal, optical (UV light), X-ray, radioactive (gamma radiation), nuclear, and microwave radiation. To generate an NTP, the amount of energy introduced into gas must be well dosed to avoid excessive heating of the plasma. This can be easily carried out using high-voltage electrical discharges in various arrangements. The most commonly used ones are corona discharges, dielectric barrier discharges, jet discharges (also called plasma needles, plasma torches, or plasma pens) with various working gases, gliding arcs, microwave discharges, and others.

Direct current corona discharges are the simplest and most accessible type of discharge used to generate an NTP. In its simplest form, a corona discharge can be obtained by applying a high voltage between a needle and a plane electrode. Various regimes and properties of direct current discharges are presented in the paper [24]. Dielectric barrier discharge (DBD) devices consist of two electrodes, where at least one of the electrodes is isolated by a dielectric barrier. Plasma jet devices involve blowing working gas through a discharge, resulting in a plasma jet being formed at the nozzle outlet. Comprehensive reviews of specific types of electrical discharges and their properties can be found in papers [25,26,27,28].

The impact of NTP exposure on different objects can have many positive effects, such as cleaning, decontamination, disinfection, modification, activation, etc. Living tissues can also be exposed to NTP, as its action is generally harmless to tissues of higher organisms, including humans [29]. However, it is essential to avoid direct contact with the high voltage applied to the electrodes.

### 1.4. NTP Applications

NTP is being actively studied for use in a wide variety of medical applications such as disinfection [30,31], accelerating blood clotting and wound healing [32,33,34], cancer treatment [35,36,37], dental diseases [38,39], and skin disorders [40,41]. Significant advances involving NTP applications have also been reported in various industrial fields, including the food industry [42,43,44,45]. Detailed reviews of extensive NTP applications can be found in the papers [46,47]. It is noteworthy that when used properly, NTP has no or minimal adverse impacts on the physical, chemical, nutritional, and sensory attributes of various products [48,49].

NTP technologies also open up many opportunities for advancing biology and plant science [50,51,52,53,54]. Its potential applications include stimulating seed germination [55,56,57,58], degrading mycotoxins [59,60], controlling plant diseases [61], and managing insect pests in stored goods [62]. It is worth noting the work [63] demonstrating the possibility of using NTP on a semi-operational to industrial scale. This demonstrates the significant potential of NTP and the importance of further research into its capabilities to advance NTP technologies.

Numerous issues related to NTP action in seed disinfection, modification of seed surface properties, metabolomic pathways, enzymatic activity, acceleration of seed germination, and initial growth are summarized in the papers [50,51,64,65,66]. The mechanisms of NTP effect on seeds are also discussed in the book [67]. However, despite a significant number of publications, a complete understanding of all the processes occurring during NTP treatment has not yet been achieved. This requires collecting more experimental data.

Unfortunately, very few experiments have focused specifically on breaking seed dormancy using NTP [68,69,70,71,72,73,74,75,76,77]. The papers [74,77] report the successful use of NTP to break seed dormancy of alfalfa (*Medicago sativa*) and black locust (*Robinia pseudoacacia*). Seeds of both these species have a hard hydrophobic testa that prevents them from germinating. The simplest way to break the dormancy of such seeds appears to be a conventional scarification [13,16,17], i.e., mechanical or chemical disruption of the seed coat [78]. However, scarification exposes seeds to the risk of damage, contamination, and infection. Using NTP to break seed dormancy offers, among other benefits, simultaneous seed disinfection. Experiments show that NTP can disinfect seeds infected with various phytopathogenic microorganisms, including bacteria such as *Clavibacter michiganensis* subsp. *michiganensis* and *Erwinia amylovora* [79], as well as fungi *Fusarium circinatum* [80] and *Fusarium oxysporum* [81].

In the experiments with alfalfa seeds [74], the effect of NTP was examined on wild species and cultivated alfalfa (Zuzana and Vlasta varieties). The results showed a more pronounced response to NTP treatment in wild-type alfalfa seeds, with germination efficiency increasing from 21% to 30–34%. The commercial Zuzana and Vlasta cultivars, which are characterized by fairly high germination, also showed increased germination, but only by 3% (from 91% to 94%). The experiments with black locust [77] revealed significant changes in treated seeds such as surface erosion (observed by SEM), increased oxygen content (determined by EDS), improved hydrophilicity (measured by contact angle), and increased water uptake compared to untreated seeds. In addition, black locust seeds exposed to NTP for 20 min demonstrated a remarkable increase in germination efficiency, achieving 44% compared to just 6% in untreated seeds, along with notable increases in fresh weight for both seedling shoots and roots.

Due to their strong dormant state, black locust seeds are an excellent candidate for studying and developing methods to break dormancy. Previous experiments focused on breaking seed dormancy of black locust and alfalfa [74,77] used NTP generated by a modified cometary discharge in a point-to-ring electrode system. Since the properties of NTP depend on the discharge used, this study aims to explore the effect of NTP generated by fundamentally different discharges on black locust seeds.

## 2. Materials and Methods

Black locust seeds were collected at Lake Vajnory (Bratislava, Slovakia) and divided into groups of 30 seeds. Each group was exposed to one of the plasma sources (see below) for 10 or 30 min. To assess potential thermal damage to black locust seeds resulting from the NTP treatment, we monitored the surface temperature of the seeds using a FLIR E4 thermal imaging camera. The temperature was below 40 °C for all types of NTP treatment, indicating that thermal damage to the seeds was unlikely. Each group of plasma-treated seeds as well as control (untreated) seeds were then placed in separate Petri dishes and stored in the dark at room temperature. The bottom of each Petri dish was covered with three layers of filter paper moistened with 4 mL of sterile distilled water. The moisture content of the filter paper on which the seeds were placed was monitored daily, and 1 mL of sterile distilled water was added to each Petri dish to maintain proper conditions for seed growth. The number of imbibed and germinated seeds was counted on the 1st, 2nd, and 5th day after NTP treatment. Seeds were considered imbibed if their surface area increased by at least 70%, and germinated if the seed coat cracked and the radicle became visible. To determine whether the imbibition and germination results were significant, we used a two-sided binomial test with a significance level of *p* < 0.01.

### 2.1. NTP Sources Used

(a) Corona discharges

We used two different DC corona discharges in air at atmospheric pressure to treat black locust seeds: a classical corona discharge (CD) and a so-called back corona discharge (BC). Both types of corona discharge were established in a point-to-plate electrode system. A medical stainless-steel needle was used as the high-voltage point electrode and was positioned at a distance of 10 mm above the grounded plane electrode. The point electrode was connected to the negative pole of a DC high-voltage source. A commercial perforated stainless steel plate with a thickness of 0.5 mm, a perforation hole diameter of 0.5 mm, and a distance between perforation holes of 1.09 mm was used as the plane (grounded) electrode.

**Figure 1:**
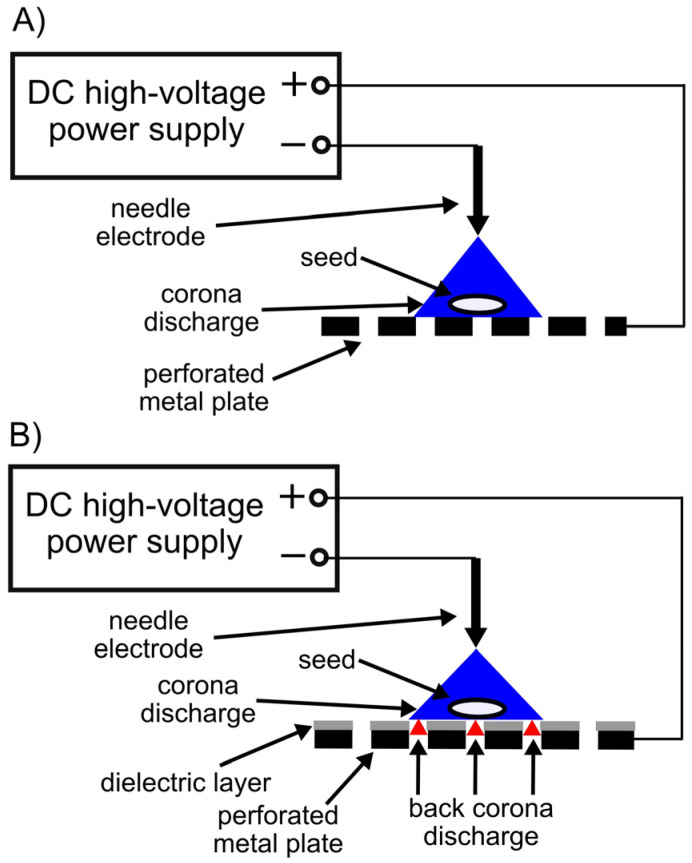
Seed treatment using the classical corona (**A**) and the back corona discharges (**B**).

The back corona discharge was obtained by coating the upper side of the grounded electrode (i.e., the perforated plate) with a dielectric layer in the form of ceramic enamel. The dielectric layer was deposited by spraying, resulting in a 120 µm thick permanent enamel layer being formed over the entire upper surface of the plane electrode. The deposited dielectric layer had a volume resistivity of 10^12^ Ω⋅m and a relative permittivity of 6. In contrast to the back corona discharge, the classical corona discharge was obtained in the same electrode system with the only difference being that the grounded perforated electrode had not been covered with a dielectric layer. Experimental setups for seed treatment using the classical corona and back corona discharges are shown in Figure 1.

Seed treatment with the corona discharges was carried out under the following conditions:

- The interelectrode distance was 10 mm;
- The discharge current was *I* = 100 μA in the classical corona discharge (CD) and *I* = 200 μA in the back corona discharge (BC);
- Treatment time was 10 and 30 min.

**Figure 2:**
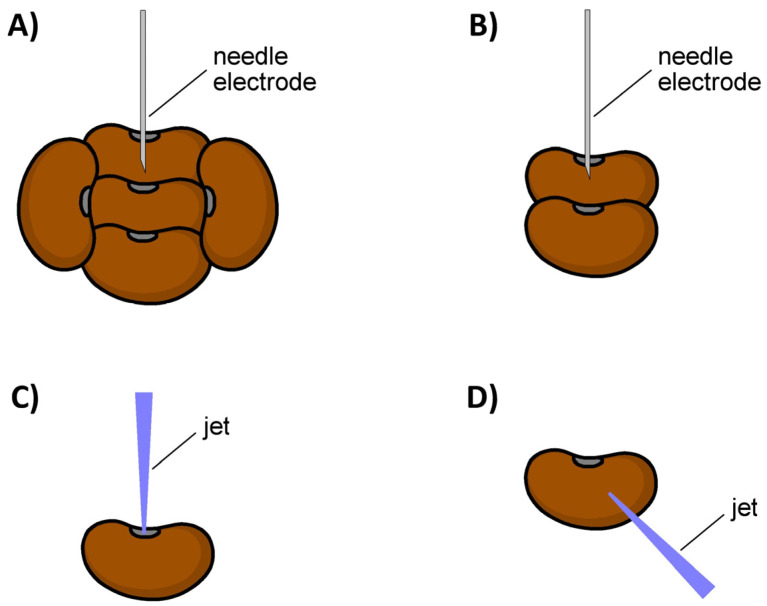
Seed placement during NTP treatment: five seeds processed simultaneously with the needle electrode placed above the central seed (**A**), two seeds processed simultaneously with the needle electrode positioned above the seed hila (**B**), a single seed treated with the argon APPJ directed to the seed hilum (**C**), a single seed treated with the argon APPJ directed to the side surface of a seed (**D**).

Seeds were treated by the corona discharges in two ways (Figure 2):

(1) Five seeds were treated simultaneously (CD-A and BC-A arrangements) as shown in Figure 2A: one seed was placed in the center and four around the central seed;
(2) Two seeds were treated simultaneously (CD-B and BC-B arrangements) as shown in Figure 2B: the needle electrode was positioned above the seed hila to target the area around the hilum to a greater extent.

(b) Atmospheric-pressure plasma jet (APPJ)

**Figure 3:**
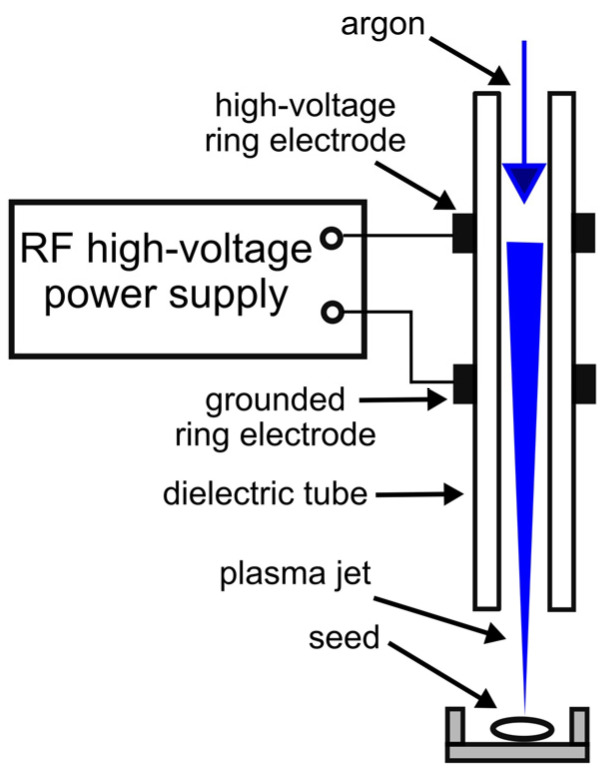
Seed treatment using an argon APPJ.

The third NTP source employed for the treatment of black locust seeds was an APPJ. The plasma jet reactor featured a ceramic tube equipped with two ring electrodes positioned on the outer surface and separated by a distance of 5 mm. The ceramic tube had an outer diameter of 5.5 mm and an inner diameter of 1.6 mm. Argon was used as the working gas to generate the APPJ, which was blown through the tube. The electrodes were powered by a high-voltage AC pulse generator (Dora PS, Wroclaw, Poland). The amplitude of the supplying voltage and its fundamental frequency were 5.5 ± 0.2 kV and 38 ± 2 kHz, respectively. The power consumed for NTP generation was regulated by setting the voltage pulse duration. A scheme of the experimental setup with the APPJ reactor is shown in Figure 3.

Seed treatment with the APPJ was carried out under the following conditions:

- Consumed power was 4.0 ± 0.2 W;
- Argon flow rate was 2.5 ± 0.2 L/min;
- The distance between the reactor nozzle and a seed was 10 mm;
- Treatment time was 10 and 30 min.

Seeds were treated with the argon APPJ in two ways (Figure 2):

(1) APPJ directed toward the seed hilum (APPJ-C arrangement) as shown in Figure 2C;
(2) APPJ directed toward the side of a seed (APPJ-D arrangement) as shown in Figure 2D.

(c) Dielectric barrier discharge (DBD)

The fourth NTP source employed for the treatment of black locust seeds was a DBD operating in air at atmospheric pressure. The DBD reactor was constructed in a planar-parallel configuration with only one electrode covered with a dielectric layer in the form of a 1 mm thick ceramic plate. The presence of the dielectric barrier between the electrodes provided a quite regular distribution of the DBD plasma in the form of short-lasting filamentary micro-discharges (the duration ranged from tens to hundreds of nanoseconds). In addition, the dielectric barrier prevented the occurrence of spark discharges in the reactor. The electrode surface area was 120 cm^2^. The air gap between the electrodes, in which the seeds were placed, was 5 mm.

**Figure 4:**
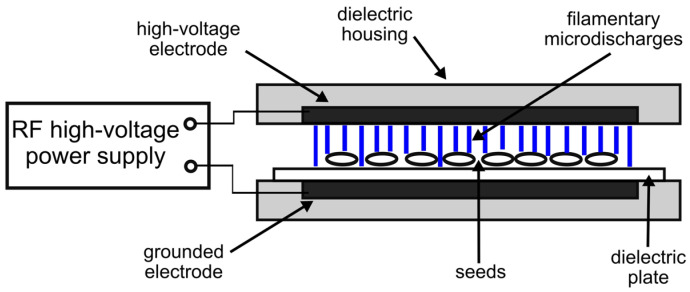
Seed treatment using a DBD.

As a power source for the DBD reactor, we used a similar high-voltage AC pulse generator as in the APPJ reactor. The operating parameters of the DBD reactor were as follows: voltage amplitude was 7.5 ± 0.2 kV, and fundamental pulse frequency was 38 ± 2 kHz. The consumed power was also controlled by modulating the duration of voltage pulses. An experimental setup with the DBD reactor is shown in Figure 4.

Seed treatment with the DBD plasma reactor was carried out under the following conditions:

- Consumed power was 15.0 ± 0.5 W;
- Treatment time was 10 and 30 min;
- All 30 seeds were treated simultaneously.

### 2.2. Characterization of the Discharges Used

**Figure 5:**
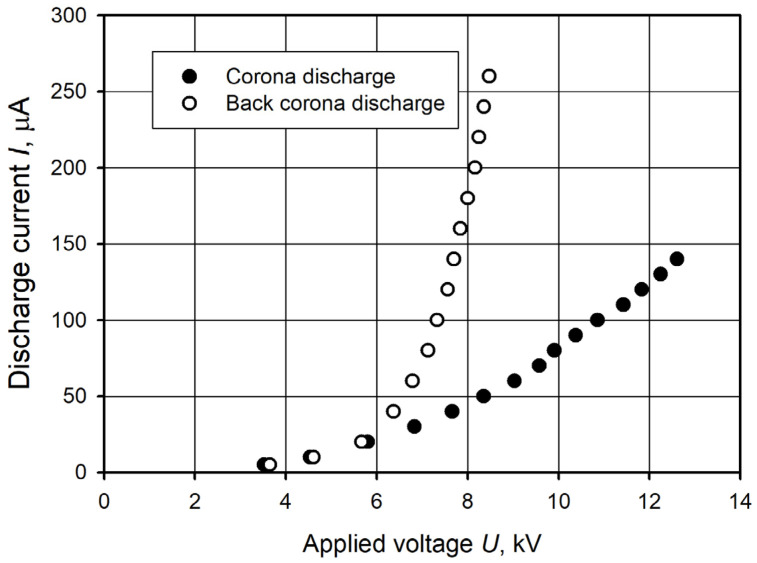
Volt-ampere characteristics of the classical corona and back corona discharges at an interelectrode distance of 10 mm.

To characterize the corona discharges, we measured the volt-ampere characteristics of both types of corona discharges used. The graphs of the volt-ampere characteristics of the classical corona and back corona discharges at the interelectrode distance of 10 mm are given in Figure 5. It can be seen from the volt-ampere characteristics of the corona discharges that the back corona discharge has a significantly higher discharge current compared to the classical corona discharge at the same interelectrode distance and supply voltage. The onset voltage (determined at a discharge current of 5 µA) was lower in the classical corona discharge and was 3.5 kV, whereas in the back corona discharge, it was 3.7 kV. With an increasing discharge voltage, a significantly steeper increase in the current was observed in the back corona discharge (Figure 5). A voltage of 10.9 kV was required to achieve a discharge current of 100 µA in the classical corona discharge, while only 7.3 kV was needed for the back corona discharge.

The higher current in the back corona discharge was due to the dielectric layer deposited on the upper side of the perforated electrode, leading to back ionization. The mechanism of the back corona discharge is as follows. Some negatively charged particles drifting from the negative needle electrode toward the positive perforated electrode accumulate on the dielectric layer. This accumulation of negative charge creates a voltage drop relative to the positive perforated electrode. When this voltage reaches the breakdown value, a back discharge occurs through the perforations, leading to the release of positive ions into the interelectrode space. These positive ions then drift toward the negative needle electrode, contributing to the overall discharge current and increasing plasma density [82,83,84]. A higher current in the back corona discharge may provide an advantage in seed treatment. Moreover, it is worth noting that the back corona discharge affects the seed surface not only from the side of the needle electrode but also from the opposite side that is in contact with the plane electrode. Black locust seeds were treated with classical corona and back corona discharges at a current of 100 and 200 µA, respectively, which corresponded to a power of 1.1 and 1.6 W.

**Figure 6:**
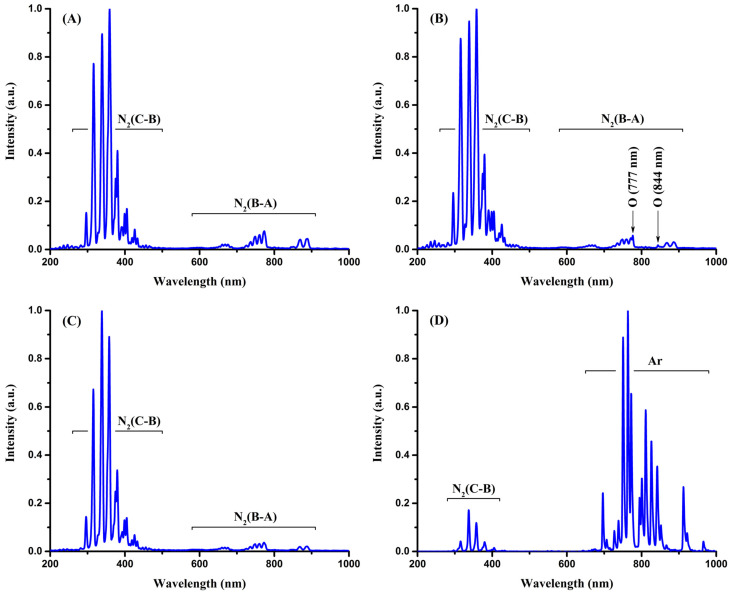
Emission spectra of the classical corona discharge (**A**), back corona discharge (**B**), DBD (**C**), and argon APPJ (**D**).

Figure 6 shows the emission spectra of the NTP sources used in the research. As one can see, the emission spectra of the classical corona (Figure 6A), back corona (Figure 6B), and DBD (Figure 6C) are very similar. They mainly consist of a low-intensity first positive system of the nitrogen molecule N2BΠg3→N2AΣu+3 in the long-wavelength region of the spectra (600–900 nm) and a high-intensity second positive system of the nitrogen molecule N2CΠu3→N2BΠg3 in the short-wavelength region (300–450 nm). The higher intensity of the second positive system is primarily due to the shorter radiative lifetime of the N2CΠu3 state [85], which results in fast and more efficient emission from this state compared to the more long-lived N2BΠg3 state. As for the first negative system of the nitrogen molecular ion N2+BΣu+2→N2+XΣg+2, only the N2+BΣu+2[υ=0]→N2+XΣg+2[υ=0] electronic–vibrational transition (λ = 391 nm) was observed in the emission spectra, which had a rather low intensity.

At wavelengths of 777 and 844 nm, low-intensity spectral lines of oxygen atoms were detected, which correspond to the 3pP5−3sS05 and 3pP3−3sS03 resonance transitions, respectively. The presence of the oxygen lines in the emission spectra indicates the production of atomic oxygen, which plays an important role in the formation of various reactive oxygen and nitrogen species such as ozone and nitrogen oxides. Although the oxygen emission lines were more distinct in the back corona discharge, the emission spectra of the classical corona, back corona, and DBD overall showed no significant differences, indicating a similar composition of these NTPs.

As far as the emission spectrum of the argon APPJ is concerned (Figure 6D), it consists of argon resonance emission lines in the long-wavelength region of the spectrum (700–1000 nm) and the second positive system of the nitrogen molecule N2CΠu3→N2BΠg3 in the short-wavelength region (300–450 nm). The presence of the argon emission lines in the spectrum is obvious because argon, being the working gas, was blown through the discharge and was directly excited by electrons. The emission of nitrogen molecules is due to Penning excitation [86,87] and occurs as follows. When exiting the nozzle, excited (metastable) argon atoms collide with nitrogen molecules present in the air. Upon collision, excited argon atoms are quenched, transferring their excitation energy to nitrogen molecules and moving them to the N2CΠu3 excited state [87,88,89,90]:

Arm∗+N2XΣg+1→N2CΠu3+Ar

The excitation of nitrogen molecules by quenching metastable argon atoms occurs due to the presence of resonant energy transfer within the N2CΠu3 excited state [87,88,89,90]. The threshold excitation energy of the N2CΠu3 state is 11.1 eV, while the excitation energies of metastable argon atoms are 11.55 eV for the P23 state and 11.72 eV for the P03 state.

Emission of the first negative system of the nitrogen molecular ion, observed in helium APPJs [91], was absent in the emission spectrum of the argon APPJ. This is due to the fact that Penning ionization is not possible in this case [92], since the excitation energy of the P23 and P03 metastable argon atoms is insufficient to ionize a nitrogen molecule, which requires 15.58 eV.

Although oxygen spectral lines were not detected in the emission spectrum of the argon APPJ, it is well established that metastable argon atoms can dissociate oxygen molecules, resulting in the production of highly reactive oxygen atoms [93]:

$${Ar}_{m}^{*}+O_{2}\to Ar+O+O$$

The production of atomic oxygen suggests that reactive oxygen and nitrogen species are also formed in the argon APPJ reactor.

### 2.3. Measurements of Water Contact Angle and Surface Free Energy

To study the effect of different NTP sources on the wettability of black locust seeds, we measured the water contact angle (WCA). The WCA was determined using a Surface Energy Evaluation System (See System 7.6, Advex Instruments, Brno, Czech Republic). Measurements were made by pipetting a 5 µL drop of sterile distilled water onto the seed surface and capturing an image of the drop with a CCD camera of the See System. The images were then processed by See System software to determine the WCA. The measurements of WCA were made for 5 seeds from each group of 30 seeds treated with different NTP sources for 30 min. Besides WCA, the See System software allows the evaluation of surface free energy (SFE), which was also used to characterize the effect of an NTP on the seed surface.

### 2.4. Analysis Using Scanning Electron Microscopy and Energy Dispersive Spectroscopy

To study the effect of different NTP sources on the morphology of the seed surface, Scanning Electron Microscopy (SEM) was employed. SEM images were obtained with TESCAN MIRA 3 LMH (TESCAN, Brno, Czech Republic) equipped with a Schottky cathode. The elemental composition of the seed surface was determined using Energy Dispersive Spectroscopy (EDS), an analytical method based on the detection of X-rays excited by the impact of an electron beam on the seed surface. In this case, an EDS spectrometer (Bruker Quantax 200, Bruker, Brno, Czech Republic) with an accelerating voltage of 10 kV, which is part of the electron microscope, was used. Seed samples were mounted on aluminum specimen stubs using double-sided adhesive carbon tape. To prevent surface charging, the seeds were coated with a 6 nm thick layer of gold. All measurements were repeated three times.

## 3. Results

### 3.1. Imbibition and Germination of Black Locust Seeds

**Figure 7:**
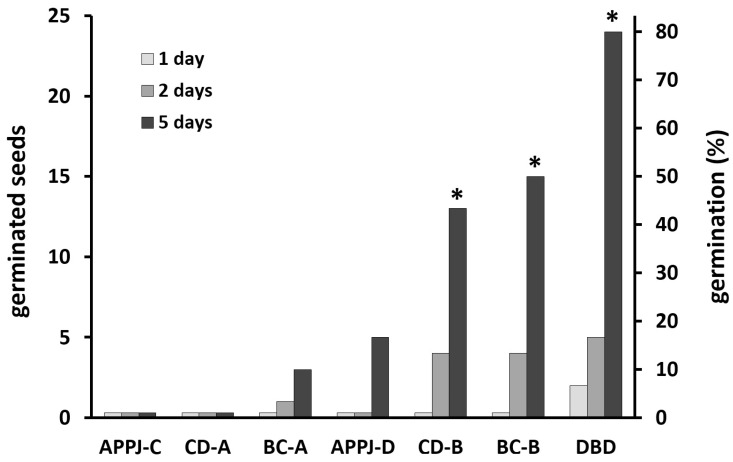
Cumulative germination of black locust seeds treated with different NTP sources for 30 min (columns marked with an asterisk indicate significant results compared to control, *p* < 0.01).

The effect of each type of NTP treatment on black locust seeds was quantified by evaluating imbibition and germination expressed as a percentage of the imbibed/germinated seeds (the total number of seeds in a given treatment was 30). These evaluations were made three times: on the first, second, and fifth day after NTP treatment. Significant results for imbibition and germination were determined using a two-sided binomial test. Control (untreated) seeds were measured each time the effect of another NTP source was tested to make sure that the conditions were the same. The results of imbibition and germination of black locust seeds treated with different NTP sources are summarized in Table 1. The dynamics of seed germination after treatment with different NTP sources for 30 min is further graphically presented in Figure 7.

According to the obtained results, it can be stated that the classical corona discharge, back corona discharge, argon APPJ, and DBD have a positive effect on both imbibition and germination of black locust seeds. However, the efficiency of different treatment methods varies significantly. In the case of the control samples (untreated seeds), none of the seeds germinated as of the fifth day after being placed in a moistened Petri dish, and the maximum number of imbibed seeds due to water uptake was equal to four, which is 13.3% of the total number of seeds in the control group.

The data in Table 1 also indicate that the placement of seeds on the plane electrode during their treatment with the classical corona and back corona discharges is of importance. Simultaneous treatment of five seeds with the classical corona discharge (CD-A treatment) were shown to be ineffective and did not improve imbibition and germination even at the longest exposure of 30 min. The percentage of imbibed seeds on day 5 after the CD-A treatment for 10 min was 0% and after the CD-A treatment for 30 min was 6.7% (i.e., two imbibed seeds), while the germination remained 0% in both cases. As for the back corona discharge, it caused seed germination in both arrangements (BC-A and BC-B treatments) but with significantly different efficiencies. An increase in the treatment duration had a slight positive effect on both imbibition and germination. On the fifth day after the BC-A treatment for 10 min, 16.7% of the seeds had imbibed and 6.7% had germinated, compared to 23.3% and 10% for 30 min treated seeds, respectively. Placing the needle electrode centrally between two seed hila (Figure 2B) caused a significant increase in the imbibition and germination compared to the simultaneous treatment of five seeds (Figure 2A). Exposure of seeds to the classical corona discharge (CD-B treatment) for 10 min resulted in 56.7% of seeds imbibed and 40% germinated on the fifth day after treatment, whereas no effect was observed after 10 min of the CD-A treatment. A longer exposure duration of 30 min (CD-B treatment) increased the percentage of imbibed and germinated seeds to 63.3% and 43.3%, respectively. The germination process after the CD-B treatment occurred gradually during seed storage, reaching a maximum of 40% and 43.3% on the fifth day for seeds treated for 10 and 30 min, respectively. Notably, the back corona discharge had 100% efficiency in terms of imbibed seeds, indicating a significant improvement in water uptake after the BC-B treatment. It is also worth mentioning that the percentage of imbibed seeds reached its maximum on the first day after the BC-B treatment and similarly after the CD-B treatment, whereas the CD-A and BC-A treatments increased the percentage gradually during seed storage. As for the germination, it was 30% on the fifth day after 10 min of the BC-B treatment and 50% on the fifth day after 30 min of the BC-B treatment. The dynamics of seed germination were similar to those after other types of NTP treatment, i.e., a gradual increase during seed storage (Figure 7).

The use of the argon APPJ demonstrated a weak effect when directing the plasma jet toward the seed hilum (APPJ-C treatment). As of the fifth day after the APPJ-C treatment, none of the seeds germinated, regardless of the treatment duration. The percentage of imbibed seeds on day 5 after the APPJ-C treatment reached 3.3% for 10 min treated seeds and 6.6% for 30 min treated seeds. Directing the plasma jet to the side surface of a seed (APPJ-D treatment) had a much better effect. The germination on day 5 after the APPJ-D treatment reached 10% for 10 min treated seeds and 16.7% for 30 min treated seeds. Longer treatment durations gave slightly better results. The APPJ-D treatment also noticeably improved seed imbibition compared to the APPJ-C treatment. The percentage of imbibed seeds on day 5 after the APPJ-D treatment was 13.3% for 10 min treated seeds and 23.3% for 30 min treated seeds, versus 3.3% and 6.6% for the APPJ-C-treated seeds, respectively.

The DBD reactor demonstrated the best efficiency in terms of imbibition and germination of seeds for both treatment durations. For instance, on the fifth day after exposing seeds to the DBD for 30 min, all the seeds in the group had imbibed, and 80% had germinated. With a 10 min treatment, the percentage of imbibed seeds was slightly lower, reaching 86.7% of the treated seeds, but all the imbibed seeds germinated. Notably, the DBD treatment also promoted almost immediate water uptake by the seeds. A huge advantage of applying the DBD reactor was the ability to process all 30 seeds at the same time.

It is worth noting that an increase in the exposure time from 10 to 30 min did not have a significant effect on seed germination, indicating that there was no need for such a long exposure time.

### 3.2. Water Contact Angle

**Figure 8:**
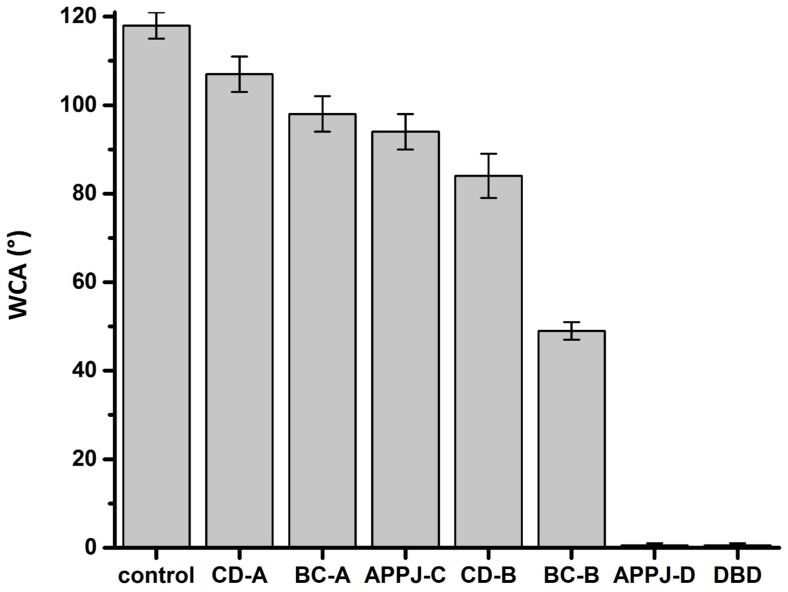
The water contact angle of black locust seeds treated with different NTP sources for 30 min.

To further investigate the effect of different NTPs on black locust seeds, we measured the water contact angle and surface free energy of the seed surface after exposure to different NTP sources for 30 min. The measurement results are summarized in Table 2. The effect of NTP on the water contact angle of seeds treated with different NTP sources for 30 min is further graphically depicted in Figure 8.

Table 2 indicates that the exposure of black locust seeds to different NTP sources for 30 min led to noticeable changes in their surface properties, expressed by water contact angle and surface free energy. The smallest changes in *WCA* and *SFE* compared to the control (untreated) seeds were observed after the CD-A treatment. The water contact angle and free surface energy of the control seeds were *WCA* = (118 ± 3) and *SFE* = (5.1 ± 0.9) mJ/m^2^, while for the seeds exposed to the classical corona discharge (CD-A treatment) for 30 min, they became *WCA* = (107 ± 4) and *SFE* = (9.2 ± 1.4) mJ/m^2^. The most significant changes in the surface properties of black locust seeds were observed after exposure to the DBD and the argon APPJ directed to the seed side surface (APPJ-D treatment). In both cases, it was not possible to measure the water contact angle, as the seed surface was completely wetted. It is noteworthy that the APPJ-C treatment, i.e., when the plasma jet was directed to the seed hilum (Figure 2C), was not as effective as the APPJ-D treatment in terms of seed surface modification. In the case of the APPJ-C treatment for 30 min, the water contact angle and surface free energy of the seed coat were equal to *WCA* = (94 ± 4) and *SFE* = (16 ± 2) mJ/m^2^. In the case of the classical corona and back corona discharges, the placement of seeds during their treatment was also of decisive importance. The results indicate (Table 2) that the simultaneous treatment of two seeds with the needle electrode positioned between them (Figure 2B) was more effective than the simultaneous treatment of five seeds with the needle electrode located above the central seed (Figure 2A). When switching from the BC-A treatment to the BC-B one, there was a significant decrease in the water contact angle from (98 ± 4) to (49 ± 2) with a corresponding increase in the surface free energy from (14 ± 2) mJ/m^2^ to (50 ± 2) mJ/m^2^, respectively. A similar trend, but less pronounced, was observed with the classical corona discharge.

### 3.3. SEM and EDS Analysis

**Figure 9:**
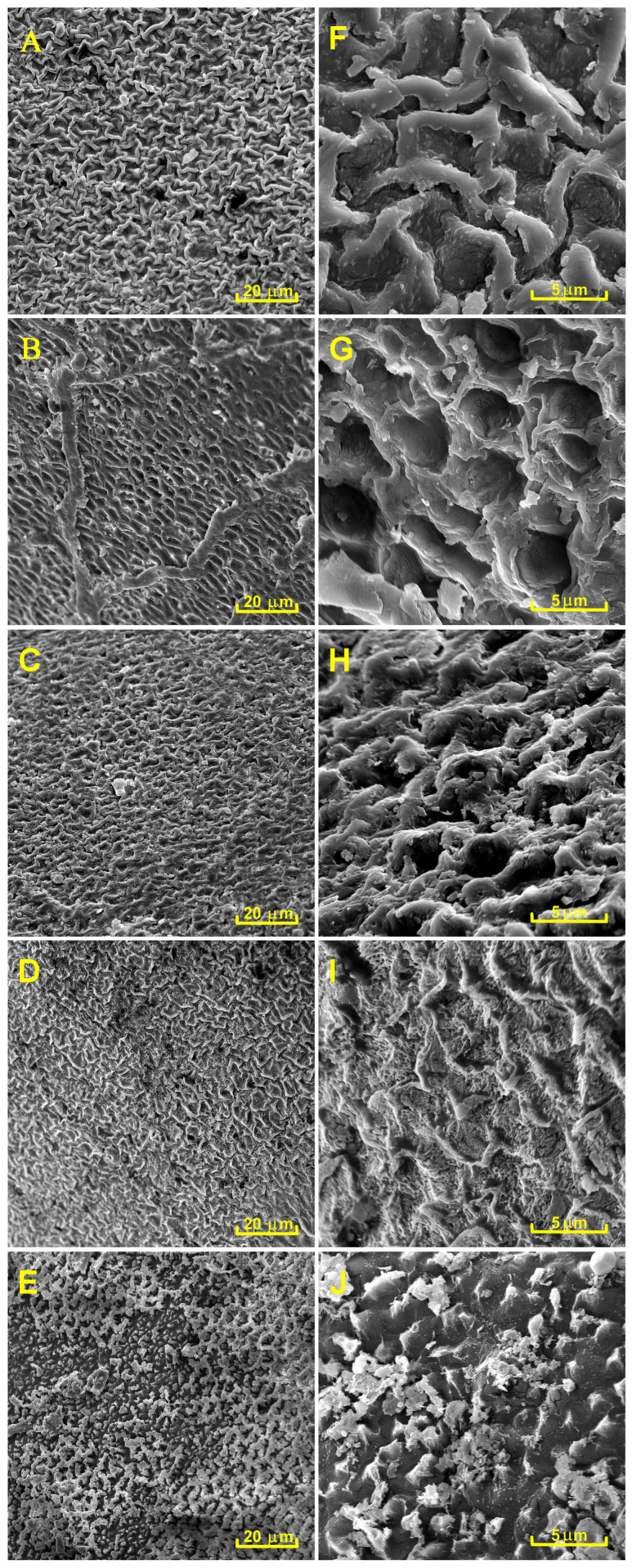
SEM images of black locust seeds treated with different NTP sources for 30 min: (**A**,**F**)—control samples, (**B**,**G**)—treated with the corona discharge, (**C**,**H**)—treated with the back corona, (**D**,**I**)—treated with the argon APPJ, and (**E**,**J**)—treated with the DBD.

To study changes in the surface morphology of black locust seeds exposed to different NTP sources, we employed SEM analysis. Due to the fact that NTP treatments in the CD-A, BC-A, and APPJ-C arrangements had no significant effect on imbibition and germination of black locust seeds (see Table 1), they were not further investigated. Figure 9 shows SEM images of black locust seeds treated with different NTP sources for 30 min, presented at two magnifications: 2000× and 10,000×.

SEM analysis showed that the classical corona and back corona discharges caused visible changes in the surface morphology of black locust seeds, which was particularly evident at higher magnification (Figure 9G,H). Exposure of seeds to the argon APPJ caused even more pronounced surface damage (Figure 9D,I). However, the most severe impact on the seed surface was caused by the DBD treatment, resulting in total destruction of the seed surface (Figure 9E,J).

**Figure 10:**
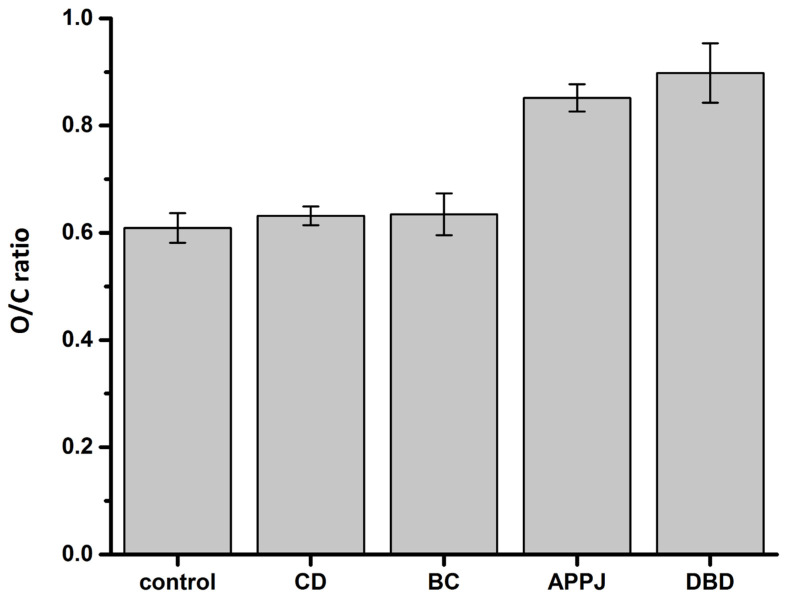
The ratio of oxygen to carbon content in the surface layer of black locust seeds treated with different NTP sources.

It is well known that air NTP can oxidize the surface of treated materials, serving as one of the primary mechanisms through which NTP alters material properties. To assess the changes in oxygen content in the surface layer of black locust seeds treated with different NTP sources, we conducted EDS analysis. Figure 10 shows the ratio of oxygen to carbon content in the surface layer of treated seeds compared to untreated (control) seeds. As one can see, the classical corona and back corona discharges had no significant impact on oxygen content in the surface layer of the treated seeds. In contrast, exposure of seeds to the argon APPJ and DBD significantly increased oxygen content, indicating pronounced oxidation of the seed surfaces.

## 4. Discussion

Seeds of black locust studied in this research have a hard testa coated with a hydrophobic wax layer that prevents water uptake, keeping seeds in a strong dormant state for a long period of time [13,14,15,16]. Šerá et al. [77] reported overcoming the dormancy of black locust seeds by treating them with a modified cometary discharge in a point-to-ring electrode configuration. The primary disadvantage of this technique is that seeds are treated individually (one at a time), necessitating precise positioning for each seed.

In the present study, we have demonstrated that the dormancy of black locust seeds can be overcome using various NTP sources. Our findings show that the classical corona discharge, back corona discharge, argon APPJ, and DBD all enhance the germination of black locust seeds, although with significantly varying efficiencies. The most effective seed germination was observed using the DBD, followed by the back corona discharge, and then the classical corona discharge. The argon APPJ demonstrated the least impact on seed germination. Importantly, unlike the cometary discharge [77], the classical corona discharge, back corona discharge, and particularly DBD allow for the simultaneous treatment of multiple seeds without the need for meticulous positioning. Therefore, the use of these NTP sources provides a significant advantage over the cometary discharge technique.

It is generally accepted that reactive oxygen and nitrogen species are primarily responsible for the beneficial effects of NTP on seed germination [94,95]. The emission spectra of the classical corona, back corona, and DBD showed no significant differences, indicating a similar composition of these NTPs. Therefore, the different impacts of the NTP sources on seed germination are most likely due to different absolute amounts of reactive species produced by each discharge, which appear to depend on the discharge type and its power. Indeed, one can see a correlation between power deposited into the discharge and its effect on seed germination. The DBD, which provided the best imbibition and germination of black locust seeds, had the highest power equal to 15 W, whereas the back corona discharge with a power of 1.6 W showed lower germination, and the classical corona discharge with a deposited power of 1.1 W yielded even worse germination outcomes. The argon APPJ, which had a relatively high power of 4 W but had the least effect on seed imbibition and germination, is in contradiction to this trend. This can be explained by a less efficient production of reactive oxygen and nitrogen species in the argon APPJ as the electric energy is primarily spent on the excitation and ionization of argon atoms.

It is also worth noting the role of discharge nonhomogeneity as an important factor influencing seed treatment outcomes. The DBD affects the entire seed surface, providing uniform treatment. In contrast, the classical corona discharge, although diffuse, impacts the seed surface only from one side. The back corona discharge, while also diffuse, treats the seed surface from both sides. Meanwhile, the argon APPJ exerts a more localized effect, targeting the seed surface at a specific point. As a result, seed treatment occurs unevenly across the surface and varies depending on the type of discharge used. In addition, the irregular thickness of the wax layer and the specific shape of each seed further contribute to uneven seed treatment. While this nonuniformity poses no significant issues when the treated seeds are planted into the soil, since water will penetrate the seeds regardless of the presence of poorly treated areas, it becomes problematic when seeds are placed with the poorly treated side on flat, water-soaked paper. In this case, water uptake by the seeds is limited, which suggests that the imbibition and germination outcomes observed in our experiments may be somewhat underestimated.

The SEM analysis revealed that different discharges have different effects on the surface of black locust seeds. Untreated seeds have a solid wavy surface without pores. In contrast, samples treated with the classical corona discharge had a noticeable disruption of the wavy structure with the formation of small “deep holes” in the wax layer. Although most of the original surface structure and its high hydrophobicity were preserved, these holes facilitated water penetration through the wax layer, initiating seed imbibition. Similar but more pronounced observations were noted in seeds treated with the back corona discharge. Notably, the EDS analysis of seeds treated with the classical corona and back corona discharges showed no significant changes in the O/C ratio compared to the control samples, indicating that overall, the surface of the treated seeds was not substantially oxidized.

In contrast to the corona discharges, the argon APPJ etched the wax layer gradually and evenly at the targeted point, without creating holes. The measurements of the water contact angle indicate that this type of NTP treatment makes the seed surface highly hydrophilic. The EDS analysis revealed a significant increase in oxygen content on the surface of the treated seeds, indicating that both the etching of the seed surface and its hydrophilization were caused by surface oxidation. Unfortunately, although the wax layer was noticeably damaged, its integrity remained intact enough to hinder water penetration. Apparently, the treatment of black locust seeds with the argon APPJ requires either increased deposited power or a much longer treatment time to completely etch the wax layer. Additionally, when factoring in argon consumption, the argon APPJ may not be the most efficient method for breaking the dormancy of black locust seeds.

The best seed imbibition and germination were achieved in the DBD, which is known to be a powerful and homogeneous NTP source. Its effect on the black locust seeds was similar to that of the argon APPJ, uniformly oxidizing and etching the wax layer without creating holes in it. However, in contrast to the localized effect of the argon APPJ, the DBD provided uniform treatment across the entire surface of the seeds. In addition, although water contact angle measurement and EDS analysis showed results similar to those of the argon APPJ, SEM analysis indicated complete etching of the protective wax layer, allowing the seeds to effectively absorb water.

Of interest is the relationship between imbibed and germinated seeds. As of the fifth day after NTP treatment, imbibition occurred in all seeds treated with the DBD and BC-B for 30 min. Notably, the ratio of germinated to imbibed seeds was significantly higher for seeds treated with the DBD (approximately 4:5), whereas for seeds treated with the BC-B, this ratio was only 1:2. Although the CD-B treatment resulted in much lower imbibition, it had a high germination-to-imbibition ratio comparable to that of the DBD treatment. The APPJ-D treatment appeared to have a similar trend, but the small number of germinated and imbibed seeds prevents a firm conclusion. Based on the obtained results, the following can be speculated. It is known that NTP treatment can stimulate the activity of enzymes involved in nutrient uptake and metabolism, such as nitrate reductase, phosphate-solubilizing enzymes, and various hydrolytic enzymes [96]. This stimulation can enhance nutrient uptake and increase nutrient availability for plant growth and development. Therefore, we hypothesize that NTP treatment of seeds not only improves their wettability and imbibition but also enhances enzymatic activity. This aligns with our previous research [77], in which we observed a similar increase in the germination of black locust seeds treated with a cometary discharge, albeit with slightly lower values. However, it is important to note that high levels of reactive oxygen species produced during plasma treatment can cause oxidative damage to enzymes, potentially leading to their denaturation or inactivation [97].

It is worth noting that although our research primarily focused on modifying the waxy seed coat with various NTP sources, overcoming seed dormancy is a much more complex issue requiring a more in-depth study of the physiology and anatomy of black locust seeds (see, e.g., [98,99]). In particular, Karaki et al. [99] showed that water uptake by black locust seeds is uneven across the seed surface, occurring primarily through specific water gaps located in the seed strophioles. Notably, the hilum and micropyle do not facilitate water uptake. These findings may help explain the varying effectiveness of different types of NTP treatment in overcoming the dormancy of black locust seeds. However, although this hypothesis may yield interesting conclusions, it can currently only serve as a basis for future studies.

## 5. Conclusions

The classical corona discharge, back corona discharge, argon atmospheric-pressure plasma jet, and dielectric barrier discharge were tested for their effectiveness in overcoming the dormancy of black locust (*Robinia pseudoacacia* L.) seeds. The findings demonstrate that all NTP sources increase seed surface hydrophilicity and enhance seed imbibition and germination. The most effective NTP source was the DBD, which resulted in 100% of black locust seeds imbibed and over 80% germinated, far outperforming the control group, where no germination occurred. The NTP sources tested modified the seed surface in two ways. The classical corona and back corona discharges created small, deep holes in the wax layer, facilitating water penetration through the holes, while retaining most of the original seed surface structure. In contrast to the localized effects of the corona discharges, the argon APPJ and DBD acted as a uniform etching agent, gradually oxidizing and hydrophilizing the seed surface. However, the argon APPJ did not completely etch the wax layer, allowing it to limit water penetration. In contrast, the DBD had the strongest effect, completely destroying the protective wax layer, which promoted efficient water uptake, making the DBD the most effective plasma source for seed treatment. The obtained results confirm the potential for using NTP treatment as a safe and effective method for overcoming seed dormancy.

## Figures and Tables

**Table 1:** Imbibition and germination of black locust seeds treated with different NTP sources (values marked with an asterisk indicate significant results compared to control, *p* < 0.01).

Type of NTP Treatment	Number of Imbibed/Germinated Seeds out of Total 30 Seeds in Each Group								
	Control			10 Min Treatment			30 Min Treatment		
	1 Day	2 Days	5 Days	1 Day	2 Days	5 Days	1 Day	2 Days	5 Days
APPJ-C	0/0	0/0	2/0	1/0	1/0	1/0	2/0	2/0	2/0
CD-A	0/0	0/0	4/0	0/0	0/0	0/0	0/0	1/0	2/0
BC-A	0/0	0/0	4/0	1/0	3/1	5/2	2/0	5/1	7/3
APPJ-D	0/0	0/0	2/0	0/0	2/1	4/3	0/0	2/0	7/5
CD-B	0/0	0/0	4/0	16 */0	16 */5	17 */12 *	17 */0	19 */4	19 */13 *
BC-B	0/0	0/0	4/0	30 */0	30 */4	30 */10 *	30 */0	30 */4	30 */15 *
DBD	0/0	0/0	4/0	22 */2	26 */4	26 */26 *	25 */2	27 */5	30 */24 *

**Table 2:** WCA and SFE of seeds treated with different NTP sources for 30 min.

Type of NTP Treatment	*WCA*, °	*SFE*, mJ/m^2^
Control	118 ± 3	5.1 ± 0.9
CD-A	107 ± 4	9.2 ± 1.4
BC-A	98 ± 4	14 ± 2
APPJ-C	94 ± 4	16 ± 2
APPJ-D	below detection limit	beyond detection limit
CD-B	84 ± 5	21 ± 4
BC-B	49 ± 2	50 ± 2
DBD	below detection limit	beyond detection limit

## Data Availability

All data are available upon request from the authors.
